# Use of high throughput amplicon sequencing and ethidium monoazide dye to track microbiota changes in an equol-producing menopausal woman receiving a long-term isoflavones treatment

**DOI:** 10.3934/microbiol.2019.1.102

**Published:** 2019-03-22

**Authors:** Lucía Guadamuro, M. Andrea Azcárate-Peril, Rafael Tojo, Baltasar Mayo, Susana Delgado

**Affiliations:** 1 Department of Microbiology and Biochemistry of Dairy Products, Instituto de Productos Lácteos de Asturias (IPLA-CSIC), Paseo Río Linares s/n, 33300-Villaviciosa, Asturias, Spain; 2 Department of Medicine, Division of Gastroenterology and Hepatology, and Microbiome Core Facility, School of Medicine, University of North Carolina (UNC), Chapel Hill, NC 27599-7555, USA; 3 Gastroenterology Department, Cabueñes University Hospital, 33203-Gijón, Asturias, Spain

**Keywords:** gut microbiota, isoflavones, equol, pyrosequencing, ethidium monoazide, menopause, metabotype

## Abstract

This work describes the impact of long term consumption of an isoflavone-rich dietary daily supplement on the composition and diversity of the faecal microbiota of a menopausal, equol-producing woman. Sequencing of 16S rDNA amplicons was performed on faecal samples taken at 0, 1, 3 and 6 months of treatment. Additionally, and for comparative purposes, ethidium monoazide (EMA) was used to avoid detection of DNA from dead bacteria. Members of two genera of the family Coriobacteriaceae (*Eggerthella* and *Collinsella*) were found in greater proportions at all sampling points during isoflavone supplementation. Different genera of the family Ruminococcaceae (e.g., *Ruminococcus* and *Faecalibacterium*), as well as members of the family Lachnospiraceae (*Coprococcus*) also underwent significant increases. For this last genus a positive correlation with the levels of equol excretion in urine was found. Currently bacterial strains known to be involved in isoflavone metabolism and equol production have been assigned to these taxa. The use of EMA dye allowed us to unravel those bacterial gut linages (e.g., Lachnospiraceae) that were more susceptible to damage. Our study contributes to the identification of microorganisms possibly involved in the production of isoflavone-desirable metabolites (such as equol), which could ultimately be isolated and further used as probiotics by people who cannot naturally benefit from isoflavones.

## 1. Introduction

Isoflavones are plant-derived phytoestrogens, belonging to the chemical family of polyphenols, present in especially large amounts in soy and soy-derived products. Isoflavones bind to oestrogen receptors, triggering physiological responses that might influence human health [1]; certainly, they have been reported beneficial in counteracting the hot flushes and vasomotor reactions experienced by menopausal women [2],[3]. However, the European Food Safety Authority (EFSA) has not substantiated health claims about the role of isoflavones in these body functions [4]. In soy, isoflavones are mostly conjugated with sugars, forming isoflavone glycosides, which have low availability and low bioactivity [5],[6]. To be fully active, isoflavone glycosides need to be transformed into isoflavone aglycones via enzymes produced by the intestinal tissue and gut microorganisms [7]. Some intestinal bacteria metabolize isoflavone aglycones into other active compounds, such as equol [7],[8]. Formed from the aglycone daidzein, equol is the isoflavone-derived metabolite with the strongest oestrogenic and antioxidant activities [9]. However, only 25–30% of Western individuals have been reported to be able to convert daidzein into equol [10] and thus, they could be the ones that harbour the appropriated gut microbiota required for its bioconversion. Although the full range of intestinal bacteria involved in equol formation remains unknown [5],[11], most of those recognised as equol-producers to date are members of the family Coriobacteriaceae [12].

As polyphenols in general, isoflavones are metabolized by components of the microbiota, and at the same time, they could also modulate the composition and/or activity of intestinal populations [13]. Analysis of intestinal microbiota modifications after isoflavone consumption could give clues on the microorganisms involved in its metabolism. Changes in the faecal community after one week of isoflavone ingestion in postmenopausal women have been previously examined [14]. However, the microbial changes that occur during long-term isoflavone intake in menopausal women have not been determined by high throughput sequencing methods. The few studies that reported on intestinal microbiota changes induced by long-term isoflavone consumption have used other culture-independent techniques including PCR-TGGE, qPCR or FISH [15]–[17], which are unable to determine low abundance microorganisms like those involved in equol production.

This study aimed to determine changes in the intestinal microbial communities during a 6-month follow up period of daily isoflavone consumption in a menopausal, equol-producing woman that was selected from previous studies [17], based on her metabolic phenotype (equol-producer) or metabotype. Additionally, we intended to know if potential isoflavone-activating microorganisms are viable in faecal samples, which may allow ultimately their cultivation and isolation. To achieve this, we treated samples with ethidium monoazide (EMA) that enters cells with damaged membranes and binds covalently to their DNA, preventing any subsequent amplification [18].

## 2. Materials and methods

### 2.1. Ethical statement, sample donor, and supplementation regimen

Ethical approval for this study was obtained from the Bioethics Subcommittee of the Spanish Research Council (*Consejo Superior de Investigaciones Científicas* or *CSIC*) and the Regional Ethics Committee for Clinical Research of the Health Service of Asturias (*Servicio de Salud del Principado de Asturias*), in compliance with the Declaration of Helsinki. Faecal samples were provided, with written consent, by a woman volunteer recruited during a previous study [17] at the Gynaecology and Obstetrics Unit (in collaboration with the Gastroenterology Department) at Cabueñes University Hospital (Gijon, Spain). This woman was selected, from a group of women identified with an equol-producing metabotype, based on her consistent phenotype and in the stability on the levels of equol production [17]. She declared having no intestinal disorders or underlying diseases, although she reported suffering menopause-related symptoms. She had undergone no medical treatment -including the taking of antibiotics- in the six months prior to beginning isoflavone supplementation, nor was any such treatments followed during the supplementation period. The woman also reported to consume a normo-type, Mediterranean diet and did not start to follow vegetarian, vegan or special dietary treatments during this period. Isoflavone supplementation consisted on a daily oral intake (80 mg/day) of a commercial dietary supplement (Fisiogen; Zambon, Bresso, Italy) rich in soy isoflavones (55–72% genistin/genistein, 28–45% other isoflavones).

### 2.2. Sample collection and processing

The volunteer provided samples of faeces (for molecular analysis) and urine (for isoflavone determinations) before treatment (basal, T0) and after one (T1), three (T3) and sixth (T6) months of isoflavone supplementation. Fresh stools were collected in sterile plastic containers and kept under anaerobic conditions in jars containing Anaerocult A (Merck, Darmstadt, Germany) for transport to the laboratory within two hours. Morning urine samples were collected by the volunteer and stored at −20 °C until analysis as previously reported [17].

### 2.3. Determination of urinary isoflavone metabolites

For the quantifications of the levels of urinary excretion of equol, daidzein and genistein, urine samples were treated as previously reported [17] and analysed by a method involving ultra-high performance liquid chromatography (UHPLC)/fluorescence detection as previously described [19].

### 2.4. Total bacterial DNA extraction

Faecal samples (0.2 g) were suspended in 1.8 mL of phosphate buffered saline (PBS) (pH 7.4) with 6.7% sucrose. These suspensions were homogenized and centrifuged at 800 rpm for 5 min at 4 °C to eliminate pieces of insoluble material and intestinal cells, and the supernatants transferred to new tubes. These were then centrifuged again, at 14000 rpm for 5 min at 4 °C. Pelleted cells were suspended in 1 mL of cold PBS and divided into two fractions: one (0.5 mL) was kept on ice until use, and the other (0.5 mL) incubated with 100 μM EMA (Biotium, Hayward, CA., USA) in the dark for 5 min with occasional mixing. The latter samples were then exposed to a 650 W halogen light source for 5 min to induce DNA crosslinking; for this, the tubes were laid horizontally on ice on a shaker platform at about 20 cm from the light source. These cells were then washed twice with cold PBS prior to DNA extraction.

Both the EMA-treated and non-treated cells were then lysed in an enzyme solution containing 20 mM TRIS-HCl pH 8.0, 2 mM EDTA, 1.20% Triton X-100, 20 mg mL-1 lysozyme (Merck) and 20 U mutanolysin (Sigma-Aldrich, Saint Louis, MO., USA). Total bacterial DNA was extracted following the protocol described previously [20], and purified using the QIAamp DNA Stool Minikit (Qiagen, Hilden, Germany). Finally, the DNA was eluted in 100 µL sterile molecular grade water (Sigma-Aldrich), and its concentration and quality determined using an Epoch microvolume spectrophotometer (BioTek Instruments, Winooski, VT., USA).

### 2.5. Library construction and pyrosequencing

A segment of the 16S rRNA genes from the purified total bacterial DNA (both from the untreated and EMA-treated samples) was PCR-amplified using the universal primers Y1 (5′-TGGCTCAGGACGAACGCTGGCGGC-3′) (position 20–43 on the 16S rRNA gene of *Escherichia coli*) and Y2 (5′-CCTACTGCTGCCTCCCGTAGGAGT-3′) (positions 361–338) [21]. These primers amplify a 348 bp stretch of the prokaryotic rDNA embracing the V1 and V2 hypervariable regions. 454-adaptors were included in both the forward (5′-CGTATCGCCTCCCTCGCGCCATCAG-3′) and reverse (5′-CTATGCGCCTTGCCAGCCCGCTCAG-3′) primers, followed by a 10 bp sample-specific barcode. Amplifications were performed using the NEBNext High-Fidelity 2x PCR Master Mix Kit (New England Biolabs., Ipswich, MA, USA) as follows: 95 °C for 5 min, 25 cycles of 94 °C for 30 s, 60 °C for 45 s, 72 °C for 30 s, and a final extension step at 72 °C for 5 min. The amplicons produced were purified using the GenElute PCR Clean-Up Kit (Sigma-Aldrich), and their concentration measured as described in subsection 2.4.

An amplicon library was prepared for pyrosequencing by mixing equal amounts of amplicons from the eight different samples (i.e., T0–T6 for both treatments). Pooled amplicons were then sequenced using a 1/8 picotitre plate in a 454 Titanium Genome Sequencer (Roche, Indianapolis, IN, USA).

### 2.6. Sequence and data analysis

Raw sequences were denoised and filtered out of the original dataset. Filtering and trimming were performed using the Galaxy Web Server [22], employing the sliding window method. Only reads longer than 150 bp were used in further analysis. Chimeras were eliminated using the USEARCH v.6.0.307 clustering algorithm routine in *de novo* mode [23]. After demultiplexing, high quality rDNA sequences were classified taxonomically using the Ribosomal Database Project (RDP) Bayesian Classifier [24] with an 80% confidence threshold to obtain the taxonomic assignment and relative abundance of the different bacterial groups. Genus was the lowest taxonomic level contemplated. Rarefaction analysis was performed to obtain the number of operational taxonomic units (OTUs) in each sample. This allowed different diversity indices to be calculated and comparisons made between samples [25]. Sequences at least 97% sequence similarity -the consensus species boundary threshold [26]- were clustered into OTUs using the CD-Hit clustering method [27] and employed in the generation of rarefaction curves using aRarefactWin freeware (produced by S. Holland; http://strata.uga.edu/software/). The raw data obtained in this study were deposited in the Sequence Read Archive (SRA) of the NCBI database (http://www.ncbi.nlm.nih.gov) under accession number: SRP131870.

### 2.7. Statistical analysis

For the analysis of sequencing data, the RDP Library Compare tool was used to estimate (by Northern analysis) the probability of observing differences in abundance in a given phylogenetic taxon [24]. Data of isoflavones metabolites in urine was analyzed using IBM SPSS 23 statistic software. Graphics were constructed using Microsoft Excel program and SigmaPlot software. The level of significance was set at *p* values of < 0.05. The Spearman's rho correlation coefficient was calculated, applying a false discovery rate (FDR) correction of 0.25, to elucidate the relationship between significantly affected taxonomic groups and equol excretion during the studied period.

## 3. Results

### 3.1. Urinary excretion of isoflavones metabolites after dietary supplementation

**Figure 1.**
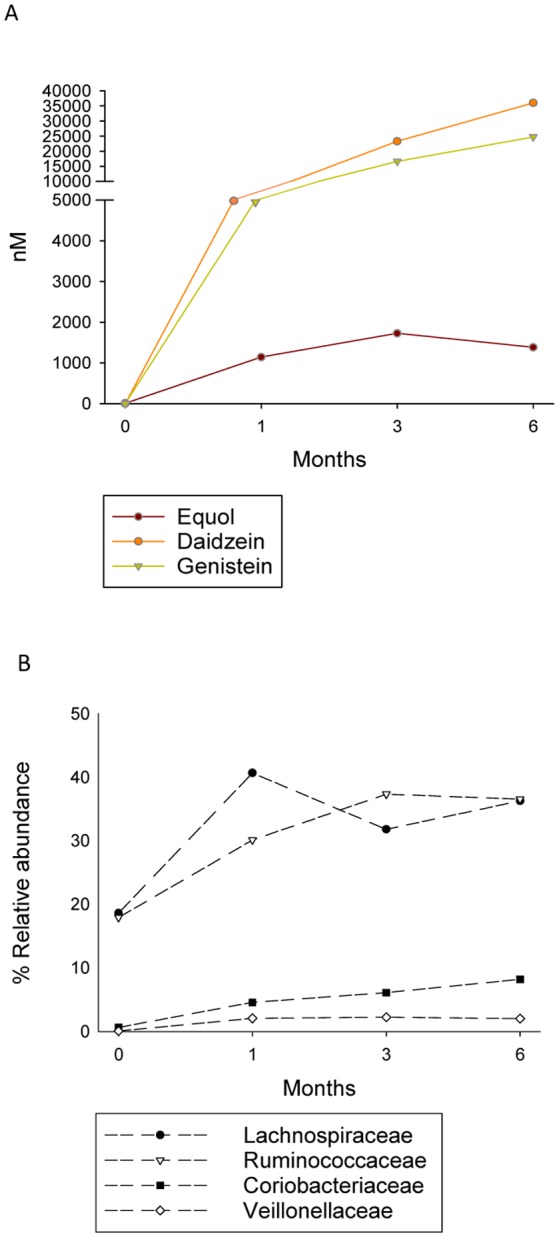
Levels (in nM) of excreted equol, daidzein and genistein in urine (A), and relative abundances (%) of significantly increased bacterial families in faeces (B) during consumption of isoflavones at different months along the interventional studied period.

In the urine samples of the woman of this study, equol concentration increased from 9 nM at baseline to 1,143 nM at T1 (Figure 1 A). Similar equol concentrations were detected at T3 and T6 (1,727 nM and 1,382 nM respectively). Daidzein and genistein in urine were absent at basal time (T0) increasing with isoflavone consumption along the studied period reaching concentrations of 23,311 and 16,620 nM respectively at T3, and 36,044 and 24,733 at T6.

### 3.2. Change in faecal microbiota over isoflavone supplementation

After denoising, performing chimera checks, and trimming the reads by length (150–400 bp), a mean of 6,030 (± 3,151) high quality sequences was obtained. Taxonomic analysis grouped the sequences mainly into five phyla: Firmicutes, Actinobacteria, Bacteroidetes, Proteobacteria and Verrucomicrobia. Fifty two genera were identified, as well as five groups of clostridia (*Clostridium* cluster IV, cluster XI, cluster XIVa, cluster XVIII and *Clostridium sensu stricto*), and two taxa with family-associated *incertae sedis* (*inc. sed.*) members (Erysipelotrichaceae *inc. sed.* and Lachnospiraceae *inc. sed.*). Taxonomic groups present at an abundance of < 0.1% were designed as ‘others’. A mean of 738 sequences per sample remained unclassified.

**Figure 2.**
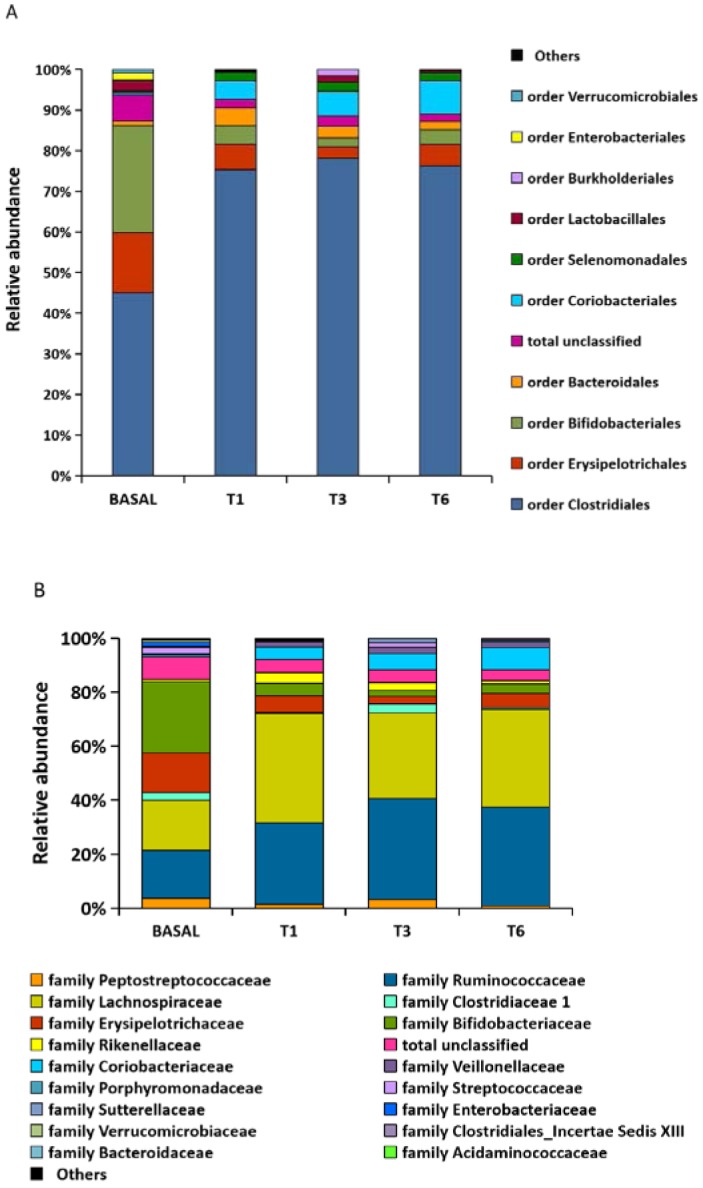
Changes in microbial composition during isoflavone consumption. The microbial composition at the order (A) and family (B) levels found in faecal samples of a menopausal woman before isoflavone treatment (basal) and at T1, T3 and T6 months is represented as relative abundancies (%).

**Figure 3.**
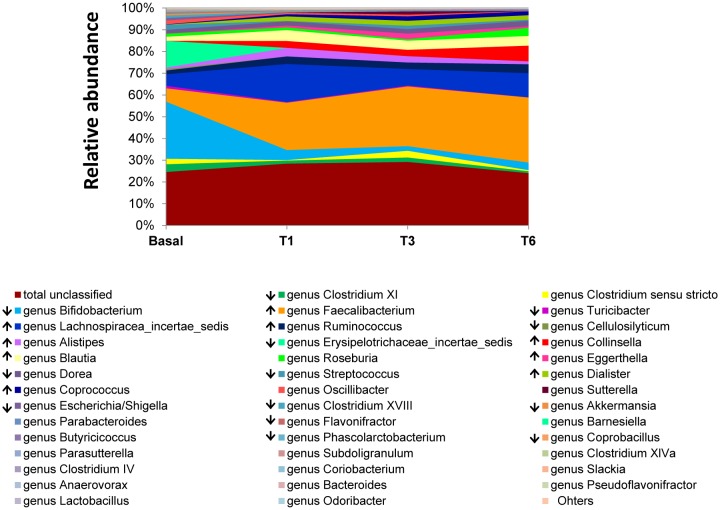
Evolution of the faecal microbiota determined at genus level. Genus composition at different time points during isoflavone treatment (Basal, T1, T3 and T6) is represented as relative abundancies (%). Genera with consistent statistical significant changes during the intervention are marked in the legend with ascending and descending arrows.

Considerable differences were observed between the bacterial communities at T0 and T1-T6. Differences were noted at the order and family levels from T1 onwards (Figure 1 B and Figure 2). A significant (*p* < 0.05) reduction in sequences was noted for 13 genera (including *Bifidobacterium*, members of Erysipelotrichaceae *inc. sed.*, *Streptococcus*, *Escherichia/Shigella*, *Turicibacter*, *Dorea*, *Akkermansia*, *Clostridium* groups XI and XVIII, *Cellulosilyticum*, *Coprobacillus*, *Flavonifractor* and *Phascolarctobacterium*) over the study period, while nine others (*Coprococcus*, *Dialister*, *Eggerthella*, *Collinsella*, *Blautia*, *Ruminococcus*, *Alistipes*, *Faecalibacterium*, and Lachnospiraceae *inc. sed.*) increased significantly (*p*<0.05) (Figure 3). Among these, a positive correlation with levels of equol excretion in urine was only found for *Coprococcus* (Spearman correlation coefficient r = 1, Table 1).

**Table 1.** Spearman correlation analysis between the levels of urinary equol excretion and significantly affected microbial taxa in faecal samples over the study period.

Taxa	Correlation coefficient rho	Sig. (2-tailed)**	FDR=0.25*
Genus			
*Coprococcus*	1.000	**0.010**	**0.019**
*Dialister*	0.800	0.200	0.058
*Eggerthella*	0.800	0.200	0.077
*Collinsella*	0.400	0.600	0.183
*Blautia*	0.200	0.800	0.212
*Ruminococcus*	0.400	0.600	0.192
*Alistipes*	0.400	0.600	0.163
*Faecalibacterium*	0.800	0.200	0.087
*Bifidobacterium*	−1.000	**0.010**	**0.010**
*Streptococcus*	−0.200	0.800	0.240
*Escherichia_Shigella*	−0.775	0.225	0.135
*Turicibacter*	−0.400	0.600	0.202
*Akkermansia*	−0.949	0.051	0.038
*Dorea*	−0.800	0.200	0.067
*Flavonifractor*	−0.800	0.200	0.096
*Coprobacillus*	−0.775	0.225	0.125
*Cellulosilyticum*	−0.632	0.368	0.154
*Phascolarctobacterium*	−0.775	0.225	0.144
*Clostridium* XI	−0.400	0.600	0.173
*Clostridium* XVIII	−0.800	0.200	0,048
Erysipelotrichaceae *inc. sed.* genera	−0.316	0.684	0.211
Lachnospiraceae *inc. sed.* genera	0.200	0.800	0.221
Family			
Ruminococcaceae	1.000	**0.010**	**0.029**
Lachnospiraceae	0.200	0.800	0.250
Coriobacteriaceae	0.800	0.200	0.106
Veillonellaceae	0.800	0.200	0.115

**Correlation is significant at the 0.01 level (2-tailed)

*False Discovery Rate (FDR) correction of 0.25. In bold, significant associations.

### 3.3. Differences in community composition after EMA treatment

**Figure 4.**
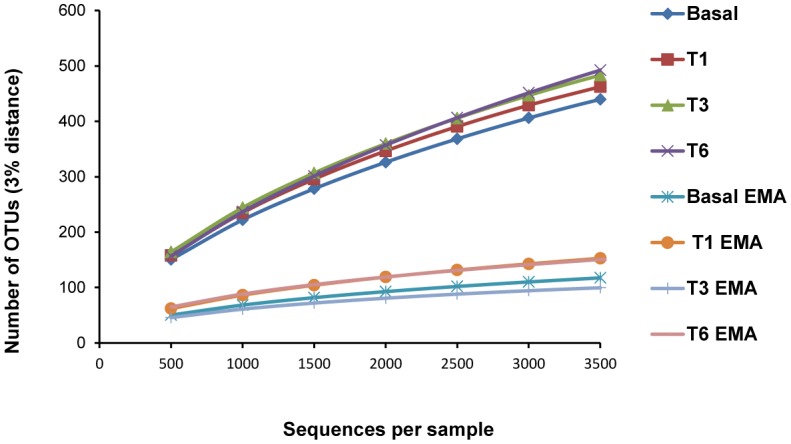
Rarefaction curves calculated for each sample treated or not with ethidium monoazide (EMA).

In the faecal samples treated with EMA before DNA extraction, the mean number of sequences recovered was higher (8,354 ± 2,932) compared to the non-treated samples (3,705 ± 408). However, the number of OTUs identified at 97% similarity was significanlty smaller, ranging from 105–244 per sample compared to 463–594 for the non-treated samples (Figure 4). The most significant and consistent reductions in reads after EMA exposition were obtained for the order *Clostridiales*, particularly for members of the family Lachnospiraceae (Table 2). Some members (*Dorea, Clostridium* cluster XIVa, *Coriobacterium* and *Sutterella*) were not even detected by treating faecal samples with this dye.

**Table 2.** Genera showing statistical differences in relative abundance (% sequences) when comparing DNA extracted directly from faeces or after exposure to ethidium monoazide (EMA).

Genera	Sample/treatment							
	Basal	Basal/EMA	T1	T1/EMA	T3	T3/EMA	T6	T6/EMA
								
***Faecalibacterium***	**6.16**	0.06***	**21.74**	1.35***	**27.46**	0.45***	**29.91**	0.80***
**Lachnospiraceae *inc. sed.***	**5.33**	0.05***	**17.23**	0.35***	**7.60**	0.52***	**10.67**	0.93***
*Streptococcus*	2.37	0.00***	0.15	0.01**	1.56	0.02***	0.53	0.04***
***Blautia***	**2.26**	0.01***	7.06	0.29***	**5.24**	0.20***	**5.50**	0.89***
*Oscillibacter*	1.81	0.00***	0.76	0.10***	0.82	0.02***	0.14	0.15
*Roseburia*	1.37	0.04***	1.26	0.17***	0.65	0.34*	3.71	0.36***
*Clostridium* cluster XVIII	1.14	0.22***	0.38	0.00***	0.08	0.00	0.07	0.00*
*Dorea*^a^	1.09	0.00***	0.32	0.00***	0.23	0.00**	0.35	0.00***
*Clostridium* cluster XIVa^a^	0.33	0.00***	0.09	0.00**	0.11	0.00*	0.16	0.00***
*Coprococcus*	0.11	0.01*	0.61	0.09***	1.79	0.04***	1.30	0.16***
***Collinsella***	**0.00**	0.00	**3.16**	0.13***	**2.93**	0.13***	**7.22**	0.30***
*Sutterella*^a^	0.00	0.00	0.09	0.00**	1.45	0.00***	0.14	0.00**
***Coriobacterium*^a^**	0.00	0.00	0.12	0.00**	0.03	0.00	0.05	0.00

*p value <0.05; ** *p* value <0.01; *** *p* value <0.001.

^a^Genera not detected by treating feacal samples with EMA before DNA extraction.

In bold, genera enriched after isoflavone ingestion, as compared to the basal sample. T1, T3 and T6 represent one, three and sixth months respectively of isoflavone supplementation.

## 4. Discussion

There is strong evidence that diet modulates the composition of the intestinal microbiota [28]. Most studies, however, have concentrated on the effect of fat and fibre [29],[30]; while other dietary microcomponents, such as polyphenols, have received less attention [31]. Certainly, little is known about the influence of isoflavones on the microbial populations of the gut [14]–[16]. The observation of general trends in the modulation of gut microbiota populations by these dietary components is hampered by the wide microbiota variations existing between subjects. In this study, with the intention of identifying relevant microbial changes over time with the ingestion of isoflavones during a long period in an individual with a particular metabotype, we selected and made use of samples from one single menopausal woman that clearly showed an equol-producing phenotype (equol excretion in urine > 1,000 nM after isoflavone challenge) according to the criterion of Rowland [32].

Although several strains of the human gut -mostly members of the family Coriobacteriaceae [12]- have been found to produce equol, the list of taxa able to do so is probably far from complete. In the present work, the abundance of two genera of this family -*Eggerthella* and *Collinsella*- significantly increased over the study period. Meanwhile *Eggerthella* has been described as capable of synthesizing equol, *Collinsella* has not been reported to produce equol [12], although increase in this genus in response to isoflavone treatment has been previously observed [14]. Other bacteria mostly belonging to the families Ruminococcaceae (*Ruminococcus*, *Faecalibacterium*) and Lachnospiraceae (*Coprococcus*, *Blautia* and *inc. sed.*) were also increased after isoflavone supplementation in our study. Enrichment of some of these bacterial gut members belonging to the Coriobacteriaceae, Ruminococcaceae and Lachnospiraceae families were already seen *in vitro* faecal cultures with isoflavones [33]. The family Lachnospiraceae has a very large presence in the human gut and has been linked to the production of butyric acid [34], a compound with several beneficial effects on the gastrointestinal ecosystem [35]. Within this family the genus *Coprococcus* is of note. It was previously shown to be abundant in faecal cultures yielding equol *in vitro* and its relative abundance in the faeces of the woman of this study correlates positively with urinary equol excretion [33].

In the woman of our study we observed an increase in Gram-positive bacteria of the phylum Actinobacteria involved in isoflavone aglycones metabolism such as *Collinsella* and *Eggerthella*, but a reduction in other members of Actinobacteria, such as *Bifidobacterium* in consonance with previous observations [18]. Further, the abundance of *Bifidobacterium* reads negatively correlated with the levels of equol in urine during the studied period. Apart from the notable reduction in this genus, sequences related to *Clostridium* clusters XI and XVIII, and those of *Escherichia/Shigella*, were also less abundant after isoflavone supplementation. Isoflavone intake may favour the development of certain bacterial types in the gut by providing an energy source. The competitive advantage might indirectly lead to the inhibition of other bacteria. Conversely, like other polyphenols, isoflavones and their metabolites have been shown to possess antimicrobial activity against specific bacterial groups [36],[37], such as members of the families Clostridiaceae and Enterobacteriaceae [38]. Different mechanisms of action have been proposed, such as the binding of these compounds to bacterial cell membranes disturbing its functioning, and via the formation of polyphenol-metal ion complexes, which could lead to iron deficiency [10].

A major advantage of culture-based identification is that it allows the isolation of the bacteria involved in isoflavone metabolism. The use of high throughput sequencing techniques, however, can help to detect low-abundance bacteria that have yet to be cultured. As previously suggested [11],[38], different bacteria may contribute towards equol production, but these might be present in the gut in low abundance, making difficult their detection by culturing.

DNA from faeces of the equol-producing woman was extracted with and without prior EMA treatment. This dye has been used in the study of microbial communities found in complex environments, such as an anaerobic digestion plants [39] and water [40]. However, in the human gut ecosystem little research has been done until now to ascertain the value of EMA for distinguishing dead from live populations. In our study, the higher number of sequences observed in samples treated with EMA might result from the interference of this compound, covalently bonded to DNA, with the spectrophotometric measure of DNA [41]. A possible underestimation of DNA concentration in the EMA-treated samples would cause a misbalance of the library, yielding a higher number of sequences. Although more sequences were retrieved in EMA-treated samples, the diversity obtained was lower, with smaller number of OTUs as compared with non-treated samples. This indicates that some microorganisms (the ones that did not amplify) have damage membranes, being probably in a no-viable state.

In our study, EMA treatment considerably altered the community sequence profile of the faecal microbiota, particularly that of the dominant anaerobic taxa such as Lachnospiraceae. This finding revealed the importance of keeping adequate (anaerobic) conditions needed to ensure protection, stability and bacterial viability in faecal samples, from stool sampling collection until handling and processing, especially when searching for the culture and potential isolation of faecal bacteria of interest, such as those producing equol or other isoflavone derived metabolites.

Although this study is focused in samples from one single individual, and more studies with different populations and metabotypes are needed to understand the effects of isoflavone intake, we think that the description of consistent specific gut microbial changes over time with the ingestion of isoflavones in a case study metabotype (equol-producer) constitutes a meaningful contribution for understanding the modulation of the gut microbiota by these polyphenols.

## 5. Conclusion

This study allowed the changes in faecal microbial communities caused by isoflavone supplementation during 6 months to be monitored in a specific metabotype (equol production phenotype). Isoflavone consumption was associated with significant increases in members of the families Coriobacteriaceae, Ruminococcaceae and Lachnospiraceae, to which strains that metabolise isoflavone and produce equol are known to belong. A positive association between equol excretion and members of the families Ruminococcaceae and Lachnospiraceae was observed. These taxa might be of interest in the search for isoflavone-metabolizing microorganisms, including equol-producing bacteria. These microorganisms, after isolation, proper testing and safety assessment, could be used as probiotics by people who cannot naturally benefit from isoflavones. For their recovery from faeces is necessary to keep faecal microbiota, including strict anaerobes, in a viable stage.
